# A Pancancer Study of PIEZO1 as a Prognosis and Immune Biomarker of Human Tumors

**DOI:** 10.1155/2022/6725570

**Published:** 2022-06-14

**Authors:** Yunhui Wu, Jingying Zhang, Can Hou, Hongyu Wang, Min Zhu, Xin Yao

**Affiliations:** ^1^Department of Respiratory & Critical Care Medicine, The First Affiliated Hospital of Nanjing Medical University, 300 Guangzhou Road, Nanjing 210029, China; ^2^Department of Cardiovascular Medicine, The First Affiliated Hospital of Nanjing Medical University, 300 Guangzhou Road, Nanjing 210029, China

## Abstract

PIEZO1, a mechanosensitive ion channel protein, has been identified in the correlation between several cancers. However, the systematic pancancer study of PIEZO1 still lacks. We examined PIEZO1 across thirty-three types of cancers to explore its role in prognosis and immunological function for the first time. Based on the open databases TCGA, GTEx and CPTAC, PIEZO1 has been demonstrated to be differentially expressed in most cancers compared to adjacent normal tissues. The distinct correlation between PIEZO1 and prognosis of tumor patients was explored by GEPIA2. Genetic alteration of PIEZO1 in the TCGA tumors showed that mutation is the alteration which is linked to OS, DSS, DFS and PFS in some tumors. Alterations of protein phosphorylation levels were detected in some cancers based on the CPTAC dataset. PIEZO1 expression was linked with immune cell infiltration, such as endothelial cell and cancer-associated fibroblast. Finally, KEGG and GO enrichment analyses were applied to investigate the molecular mechanism of PIEZO1. Our first pancancer analysis illustrated the roles of PIEZO1 in different types of tumors.

## 1. Introduction

Cancer has the highest worldwide mortality rate of any disease [1]. Malignant tumors pose a severe threat to human health. Despite advances in diagnosis and treatment, cancers still cause the extremely high morbidity and mortality. Conducting a pancancer study of some certain genes and exploring its role in some malignant tumors and their corresponding cancers are highly warranted.

Piezo-type mechanosensitive ion channel component 1 (PIEZO1) characterized as the ion-conduction subunit of mechanically activated ion channel can initiate the intracellular Ca^2+^ response [2]. PIEZO1 is widely distributed and expressed in various human organs and tissues, such as the cardiovascular system [3], brains [4], lungs [5], gastrointestinal tract [6] and bladder [7]. Moreover, recent relevant literature suggest that such expression of PIEZO1 could be different in various tumors, for example, the gastrointestinal system [8], urinary system [9], respiratory system [10] and reproductive system tumors [11].

The increasing evidences indicate that tumor immune microenvironment (TME) is strongly related to carcinogenesis and cancer progression [12]. The biomarkers associated with TEM indicating prognosis and survival may be crucial to immunotherapy.

It is commonly known that calcium signaling associated with immune cells plays a significant role in the expression of transcription factors and enzymes that regulate cancer development [13–15]. Numerous literature report that PIEZO1, recognized as a mechanical stress sensor in some immune cells, crucially relates to immune regulation [16–18]. The importance of PIEZO1 activity in immune cells has been confirmed which implies the potential contribution of PIEZO1 to cancer immunotherapies.

Despite above, there is no pancancer study exploring the correlation between PIEZO1 and different tumors, since most research studies involving PIEZO1 in tumors focus on one certain cancer. In this work, we adopt a diversity of databases such as The Cancer Genome Atlas (TCGA), Genotype Tissue-Expression (GTEx), cBioPortal, Gene Ontology (GO) and Kyoto Encyclopedia of Genes and Genomes (KEGG) to assess the expression levels of PIEZO1 and the link with survival in various tumors. We further assessed the potential correlations between PIEZO1 expression levels and genetic alteration, protein phosphorylation, immune infiltration and pathway enrichment in 33 types of cancers. Our results showed that PIEZO1 can be used to predict prognosis of different cancers, and it affected tumor infiltrating immune cells, which rises a pivotal role in tumor immunity.

## 2. Materials and Methods

### 2.1. Gene Expression Level Analysis

The mRNA levels of PIEZO1 in a variety of cancers were analyzed by Tumor Immune Estimation Resource, version 2 (TIMER2). The differential expression levels of PIEZO1 were observed between the cancerous region and normal tissue for various types of tumors. The range was set as follows: log2 fold change (log2 FC) ≥ 1 or ≤ −1 and *p* value ≤ 0.05. The Gene Expression Profiling Interactive Analysis, version 2 (GEPIA2) was employed to analyze the PIEZO1 mRNA levels in some certain tumors with no corresponding normal tissues. Additionally, violin plots were applied to reveal the relationship between PIEZO1 and pathological stages of cancers by using the “Stage Plot” of GEPIA2.

UALCAN, an interactive, user-friendly, and synthetic online platform to analyze open-source TCGA data, was taken to analyze expression levels of protein with the Confirmatory/Discovery tool of the Clinical Proteomic Tumor Analysis Consortium (CPTAC). For its part, phosphoprotein levels of PIEZO1 were evaluated between tumor and normal tissues by CPTAC analysis.

### 2.2. Prognosis and Survival Analysis

The “Survival Map” was employed to detect the overall survival (OS) and the disease-free survival (DFS) of PIEZO1 in all the tumors in TCGA. The values for dividing groups with high and low expressions were defined as cutoff-high (50%) and cutoff-low (50%) values, respectively. Survival plots were obtained from the “survival analysis” module of GEPIA2. Among these, two curves were compared by the log-rank test. Meanwhile, univariate Cox regression analysis of PIEZO1 was performed in tumors where PIEZO1 was an independent prognostic indicator.

### 2.3. Genetic Alterations Analysis

Data about PIEZO1 genetic mutation type, alteration frequency, mutated sites, and copy number were retrieved through cBioPortal [19]. The information of OS, DFS, and progression-free survival (PFS) differences between with and without PIEZO1 alteration in different tumors was obtained from the “Comparison” module. Kaplan–Meier plots with the log-rank *p* value were generated.

### 2.4. Gene Set Enrichment Analysis

To study the potential function of PIEZO1 in pancancer, we divided the samples into high and low-expressed groups based on the PIEZO1 expression in each cancer type, consisting of the top 30% and bottom 30%. Then, the gene set enrichment analysis (GSEA) (http://www.gsea-msigdb.org/gsea/index.jsp) was performed using the “clusterProfiler” R package. GSEA gene sets (H, C2, and C5 collections) were downloaded from the Molecular Signatures Database (v7.5.1). The FDR-adjusted *p* < 0.05 was considered statistically significant.

### 2.5. Immune Cell Infiltration Analysis

We utilized the “Immune-Gene” unit of TIMER2 to discover the link between the levels of PIEZO1 expression and the infiltration of immune cells, such as endothelial cells, and cancer-associated fibroblasts. For this purpose, we evaluated different cancers. The algorithms QUANTISEQ, XCELL, MCP-COUNTER, EPIC, TIMER, CIBERSORT, and CIBERSORT-ABS were applied to make immune infiltration estimations. *P* values and partial correlation (cor) values were obtained by using Spearman's rank correlation test, purity-adjusted. The heatmap and scatter plot were displayed as the outcome.

### 2.6. PIEZO1-Related Partners Enrichment Analysis

PIEZO1-binding proteins were searched by the STRING website, using the query of “PIEZO1.” Then, we used Pearson correlation to analyze the first 100 PIEZO1-targeting genes which were based on the differential expression records of TCGA tumors and normal tissues in GEPIA2. The heatmap consisted of top 5 genes, which contains the partial correlation index, and *p* value was obtained using the “Gene_Corr” component of TIMER2 by the method of the purity-adjusted Spearman's rank correlation test. A Venn diagram viewer allowed the observation of the PIEZO1-binding and interacting genes. Two sets of data were combined to perform KEGG pathway analysis.

### 2.7. Human Samples

Human samples were obtained from subjects undergoing tumorectomy in the First Affiliated Hospital of Nanjing Medical University. Ethics approval was gained through the First Affiliated Hospital of Nanjing Medical University. The cancer tissues were harvested from the region of tumor. The adjacent normal tissues were collected more than 5 cm from cancerous tissues. Eight paired lung tumor tissues, stomach tumor tissues, and colon tumor tissues, as well as corresponding adjacent normal tissues were collected.

### 2.8. Immunohistochemical Staining

All human sample slides were baked at 65°C for 2 hours and then were dewaxed and rehydrated. Slides were placed in a repair box filled with EDTA antigen repair buffer (pH 9.0, Servicebio, China, G1203), and antigen repair was conducted for 20 min at 100°C. After cooking, the slides were naturally cooled to room temperature. Then, hydrogen peroxide was used for blocking endogenous peroxidase activity. Then, blocking was performed with 10% goat serum for 1 hour at room temperature and incubated with primary antibodies PIEZO1 (Abcam, UK, ab128245, 1 : 200) diluted in blocking solution overnight at 4°C. The slides were incubated with HRP secondary antibody for 2 hours at room temperature. Finally, sections were detected by DAB staining. After stained with DAB, images of samples in the slides can be taken viewing using the microscope. Slides were washed three times with PBS between each step.

### 2.9. Cell Culture

The human pancreatic tumor cell line CFPAC was cultured in the formulation of DMEM (Gibco, American, C11995500BT) with 10% FBS (BI, Israel, 04-001-1A) and 1% penicillin/streptomycin double-resistant fluid (Gibco, American, 15140122).

### 2.10. Immunofluorescence

Cells were grown in 48-well plates. When grown to 70% confluence, the cells were transfected with PIEZO1 small interfering RNAs (siRNAs) (Applied GenePharma, China) using Lipofectamine 2000 (Invitrogen Life Technologies, USA, 11668-019). After 24 hours, cells were fixed with 4% paraformaldehyde for 20 min and the cell membrane was disrupted with 0.2% Triton X-100 (PBS configuration) for 15 min. Ki67 staining was performed by adding the primary antibody anti-Ki67 (Abcam, UK ab16667, 1 : 300) and incubated overnight at 4°C. The next day, cells were incubated with fluorescent secondary antibody for 2 hours at room temperature. After stained with DAPI for 10 minutes, the cells can be viewed using an inverted microscope. Between each step, cells were washed three times with PBS.

### 2.11. Scratch Assay

Cells were grown in 6-well plates. When grown to 100% confluence, a single scrape was made in the confluent monolayer using a 200 *μ*l pipette tip. Then, cells were cultured in DMEM with 2% FBS for 24 h after washing with PBS. The migration rate was determined by the scratch area at different time points and analyzed by the Image J software.

### 2.12. Statistical Analysis

Data are shown as the mean ± standard error of mean (SEM). The GraphPad Prism software v5.0 (GraphPad Software, USA) was performed for statistical analyses. The unpaired Student's *t*-test was used for statistical analyses between two groups.

## 3. Results

### 3.1. PIEZO1 Expression Analysis

**Figure 1:**
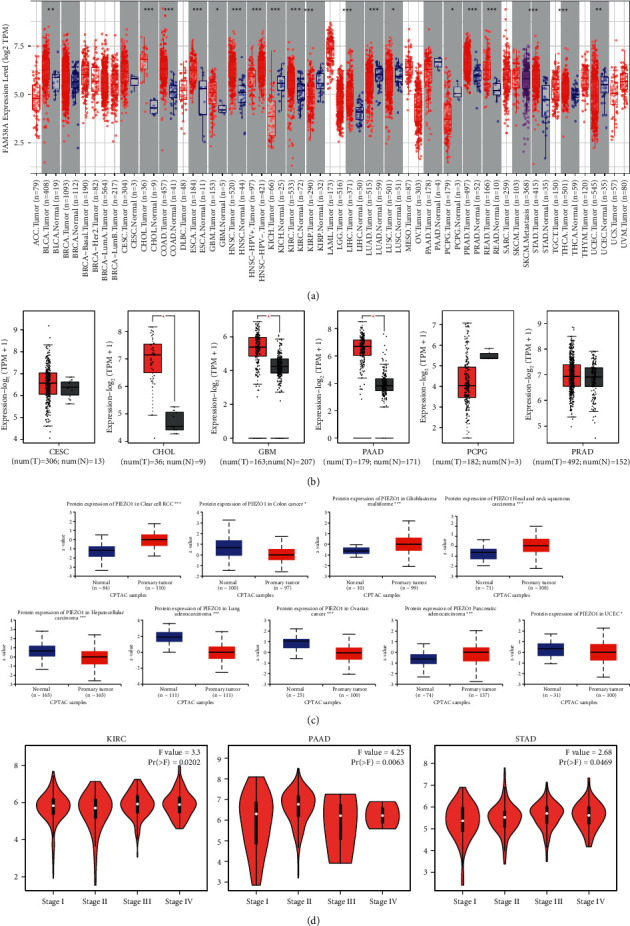
PIEZO1 expression levels in diverse human cancers and pathological stages. (a) PIEZO1 expression level in TCGA tumors analyzed by the TIMER2 database. (b) Box plot data of CESE, CHOL, GBM, PAAD, PCPG, and PRAD in TCGA cohorts compared to healthy tissues in GTEx records. (c) Protein level of PIEZO1 in normal tissue and BRCA, KIRC, COAD, GBM, HNSC, LIHC, LUAD, OV, PAAD, and UCEC. The data of protein expression were obtained and analyzed by CPTAC. (d) The expression level of PIEZO1 analyzed and compared depending on different pathological stages (stages I–IV) of KIRC, PAAD, and STAD, based on the TCGA database. ^*∗*^*P* <0.05, ^*∗∗∗∗*^*p* < 0.0001.

We first assessed the PIEZO1 expression levels in different cancers. The abbervation and full names of the 33 tumor types are given in Table 1 in Supplemental Materials. PIEZO1 expression level was higher in bladder urothelial carcinoma (BLCA) (*p* < 0.01), CHOL (*p* < 0.001), colon adenocarcinoma (COAD) (*p* < 0.001), esophageal carcinoma (ESCA) (*p* < 0.001), glioblastoma multiforme (GBM) (*p* < 0.05), head and neck squamous cell carcinoma (HNSC) (*p* < 0.001), kidney renal clear cell carcinoma (KIRC) (*p* < 0.001), liver hepatocellular carcinoma (LIHC) (*p* < 0.001), prostate adenocarcinoma (PRAD) (*p* < 0.001), rectum adenocarcinoma (READ) (*p* < 0.001), stomach adenocarcinoma (STAD) (*p* < 0.001) and thyroid carcinoma (THCA) (*p* < 0.001) and was lower in kidney chromophobe (KICH) (*p* < 0.001), kidney renal papillary cell carcinoma (KIRP) (*p* < 0.001), lung adenocarcinoma (LUAD) (*p* < 0.001), lung squamous cell carcinoma (LUSC) (*p* < 0.05), pheochromocytoma and paraganglioma (PCPG) (*p* < 0.05) and uterine corpus endometrial carcinoma (UCEC) (*p* < 0.01) than that in tumor-adjacent tissues. However, breast invasive carcinoma (BRCA), cervical squamous cell carcinoma and endocervical adenocarcinoma (CESC), and pancreatic adenocarcinoma (PAAD) showed no differential expression from tumor-adjacent tissues (Figure 1(a)). We further explored whether PIEZO1 was differentially expressed between tumor and normal tissues in the absence of data of normal tissue in TCGA. We found that cholangiocarcinoma (CHOL) (*p* < 0.05), GBM (*p* < 0.05), and PAAD (*p* < 0.05) showed higher expression in tumor tissues. No significant differences were detected between CESC, PCPG, PRAD, and their corresponding normal samples (Figure 1(b)).

In addition to the transcription level, we also obtained the protein expression level of PIEZO1 from the CPTAC dataset. PIEZO1 protein levels were significantly higher in KIRC (*p* < 0.001), GBM (*p* < 0.0001), HNSC (*p* < 0.0001) and PAAD (*p* < 0.0001) and significantly lower in COAD (*p* < 0.05), LIHC (*p* < 0.0001), LUAD (*p* < 0.0001), ovarian serous cystadenocarcinoma (OV) (*p* < 0.0001) and UCEC (*p* < 0.05) (Figure 1(c)).

We also analyzed the relationship between the PIEZO1 expression level and tumor pathological staging by GEPIA2. We found that PIEZO1 expression was changed in different pathological stages of a few tumor types, including KIRC (*p* = 0.0202), PAAD (*p* = 0.0063) and STAD (*p* = 0.0469) (Figure 1(d)).

### 3.2. PIEZO1 Expression in Tumor and Adjacent Nontumor Tissues

**Figure 2:**
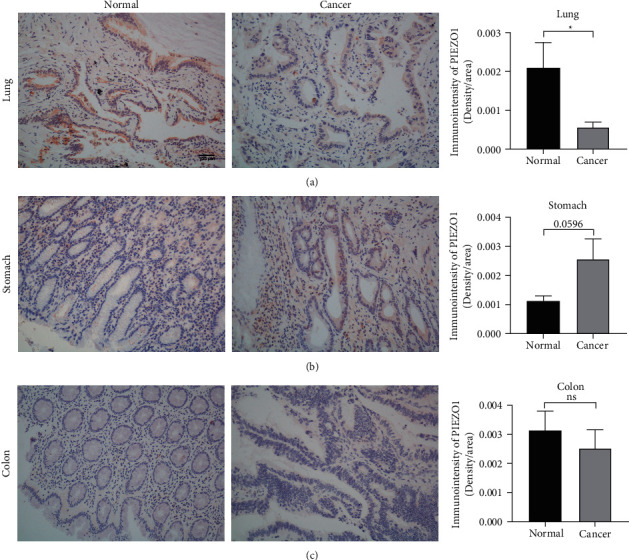
PIEZO1 expression levels in LUAD, STAD, and COAD. (a) Immunohistochemistry (IHC) staining of PIEZO1 in LUAD (*n* = 8) and adjacent normal tissue (*n* = 8) (left panel). Semiquantification of PIEZO1 positive staining (right panel). Original magnification x200. Bar = 100 *μ*m. (b) IHC staining of PIEZO1 in STAD (*n* = 8) and adjacent normal tissue (*n* = 8) (left panel). Semiquantification of PIEZO1 positive staining (right panel). Original magnification x200. (c) IHC staining of PIEZO1 in COAD (*n* = 8) and adjacent normal tissue (*n* = 8) (left panel). Semiquantification of PIEZO1 positive staining (right panel) Original magnification x200. The bar chart shown represents the mean ± SEM. ^*∗*^*P* < 0.05 versus the control group by Student's *t*-test.

Then, we detected the expression of PIEZO1 in LUAD, STAD and COAD, as well as their corresponding adjacent normal tissues by the method of IHC. Compared to adjacent normal tissues, the expression level of PIEZO1 was decreased in LUAD (*p* = 0.0389) (Figure 2(a)) and increased in STAD (*p* = 0.0596) (Figure 2(b)). There was no significant difference between COAD and its adjacent normal tissue (Figure 2(c)).

### 3.3. Survival Analysis

**Figure 3:**
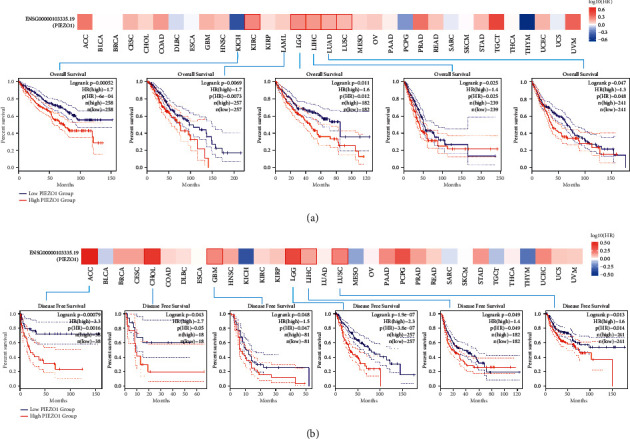
The correlation between the expression level of PIEZO1 and prognostic survival in TCGA tumors. (a) GEPIA2 utilized to analyze the relationships between the PIEZO1 expression level and overall survival (OS) of KIRC, LGG, LIHC, LUAD, and LUSC in all TCGA tumors. (b) Relationships between PIEZO1 gene expression and disease-free survival (DFS) of ACC, CHOL, GBM, LGG, LIHC, and LUHC are assessed. The survival map and Kaplan–Meier curves are presented.

Next, we studied how PIEZO1 expression correlates with prognosis and used TCGA and GEO datasets to assess OS and DFS values. Samples were divided into high-expression and low-expression groups according to the PIEZO1 expression level. The results demonstrated that among cases with KIRC (*p* = 0.0006), brain lower grade glioma (LGG) (*p* = 0.0073), LIHC (*p* = 0.012), LUAD (*p* = 0.025) and LUSC (*p* = 0.048), those with low level of PIEZO1 had longer survival time (Figure 3(a)). The data DFS analysis revealed that high expression of PIEZO1 is linked to poor prognosis for adrenocortical carcinoma (ACC) (*p* = 0.0016), CHOL (*p* = 0.05), GBM (*p* = 0.047), LGG (*p* < 0.0001), LIHC (*p* = 0.049) and LUSC (*p* = 0.014) (Figure 3(b)).

### 3.4. Genetic Alteration of PIEZO1 across Different Tumors

**Figure 4:**
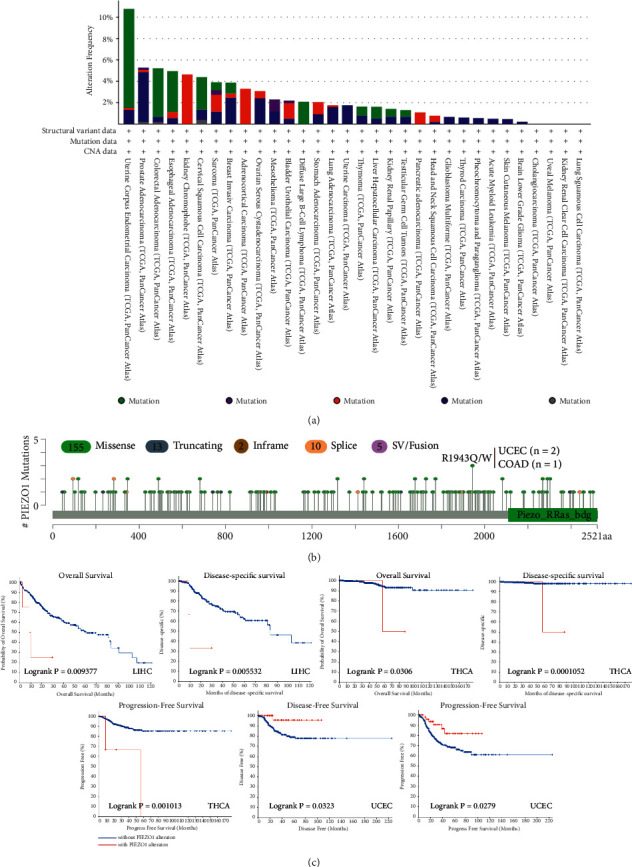
Mutation features of PIEZO1 in TCGA tumors. CBioPortal tool was utilized to explore the mutation status of PIEZO1 across TCGA tumors. (a) The type of alteration frequency. (b) The mutation site. (c) Based on the cBioPortal tool, the correlation between gene mutation and OS, disease-specific survival (DSS), DFS, and progression-free survival (PFS) of UCEC was analyzed and presented.

The oncogenesis and progression of cancers are closely associated with genomic mutations [20]. We analyzed types and sites of genetic mutation of PIEZO1 in different cancers within the TCGA database. The frequency of PIEZO1 mutation (>10%) is the highest in patients with UCEC (Figure 4(a)). In addition, the type, sites and number of the PIEZO1 genetic alteration were also discovered. The number of PIEZO1 genetic alteration is 185, which consists of 155 missense, 13 truncating, 2 inframe, 10 splice and 5 SV/fusion. R1943Q/W alteration was found in 2 cases of UCEC and 1 case of COAD (Figure 4(b)). In addition, we found the relationship between genetic mutation of PIEZO1 and survival prognosis in different types of tumors. Cases of LIHC, with PIEZO1 alteration, showed good prognosis in OS (*p* = 0.009) and disease-specific survival (DSS) (*p* = 0.006). Also, cases of THCA, with PIEZO1 alteration, had good prognosis in OS (*p* = 0.031), DSS (*p* = 0.0001) and progression-free survival (PFS) (*p* = 0.001), while alteration of PIEZO1 in UCEC was associated with worse DSS (*p* = 0.032) and PFS (*p* = 0.028) compared with no alteration (Figure 4(c)).

### 3.5. PIEZO1 Protein Phosphorylation Analysis

**Figure 5:**
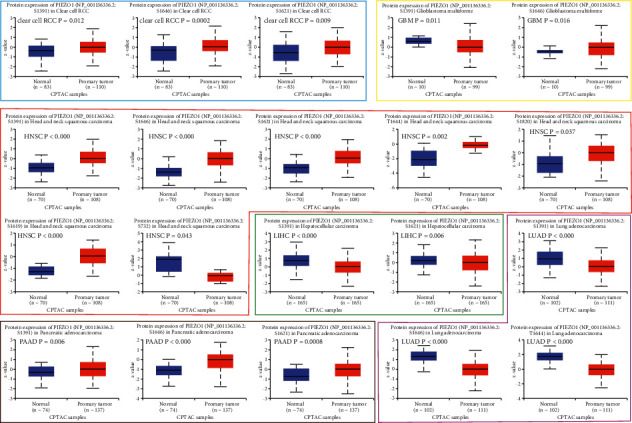
Tumor-associated protein phosphorylation of PIEZO1. (a) Box plots of specific phosphorylation sites in KIRC, GBM, HNSC, LIHC, LUAD, and PAAD.

The PIEZO1 protein phosphorylation differential levels between normal tissues and tumors were also analyzed by using CPTAC dataset. Our data presented the PIEZO1 protein phosphorylation sites and revealed the significant differences in six types of tumors (KIRC, GBM, HNSC, LIHC, LUAD, and PAAD). The S1391 site of PIEZO1 exhibits a higher level of phosphorylation in KIRC (*p* = 0.012), HNSC (*p* < 0.0001) and PAAD (*p* < 0.0001), and a lower protein phosphorylation level in GBM (*p* = 0.011), LIHC (*p* < 0.0001) and LUAD (*p* < 0.0001) compared with normal tissues, followed by a higher protein phosphorylation level of the S1646 site for KIRC (*p* = 0.0002), GBM (*p* = 0.016), HNSC (*p* < 0.0001), and PAAD (*p* < 0.0001). Our data also revealed that HNSC had the highest number of differentially phosphorylation sites, including S1391 (*p* < 0.0001), S1646 (*p* < 0.0001), S1621 (*p* < 0.0001), T1644 (*p* = 0.002), S1820 (*p* = 0.037), S1619 (*p* < 0.0001) and S732 (*p* = 0.043) (Figure 5(a)).

### 3.6. GSEA of PIEZO1 in Pancancer

**Figure 6:**
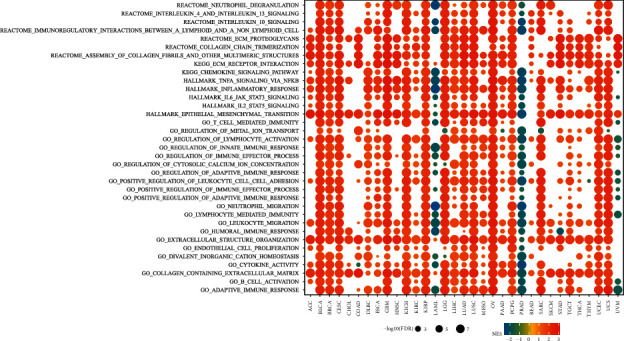
Gene set enrichment analysis (GSEA) of PIEZO1 in pancancer. (a) The size of circle represents the FDR value of enrich term in each cancer, and the color indicates the normalized enrichment score (NES) of each term.

GESA analysis was performed based on GO, KEGG, REACTOME, and HALLMARK (Figure 6). In almost all cancers, we found that PIEZO1 expression was remarkably related to immune-related pathways, such as innate immune response, adaptive immune response, inflammatory response and some inflammatory cells, such as neutrophils, lymphocyte and leukocyte. In addition, ion transport and cation homeostasis were also related to PIEZO1 in pancancers. Activation of PIEZO1 can result in calcium influx, transducing mechanical forces into biochemical responses [21]. Calcium is a ubiquitous second messenger that is responsible for a diverse host of cellular functions that are necessary for successful cancer metastasis [22]. We also found that epithelial-mesenchymal transition (EMT) hallmark was significantly enriched in high-PIEZO1 subgroups in most cancers. EMT enhances migration and invasive potential which lead to increased cancer metastasis [23]. These data revealed a potential association between PIEZO1 expression and immune activation in the tumor microenvironment (TME). In addition, the top 20 related pathways of GSEA were presented in the form of a mountain map (Supplementary Figure S1) for each type of cancer. The results showed that in most tumors, PIEZO1 was associated with the immune responses. These results further illustrated the important role of PIEZO1 in immune regulation.

### 3.7. Immune Infiltration

**Figure 7:**
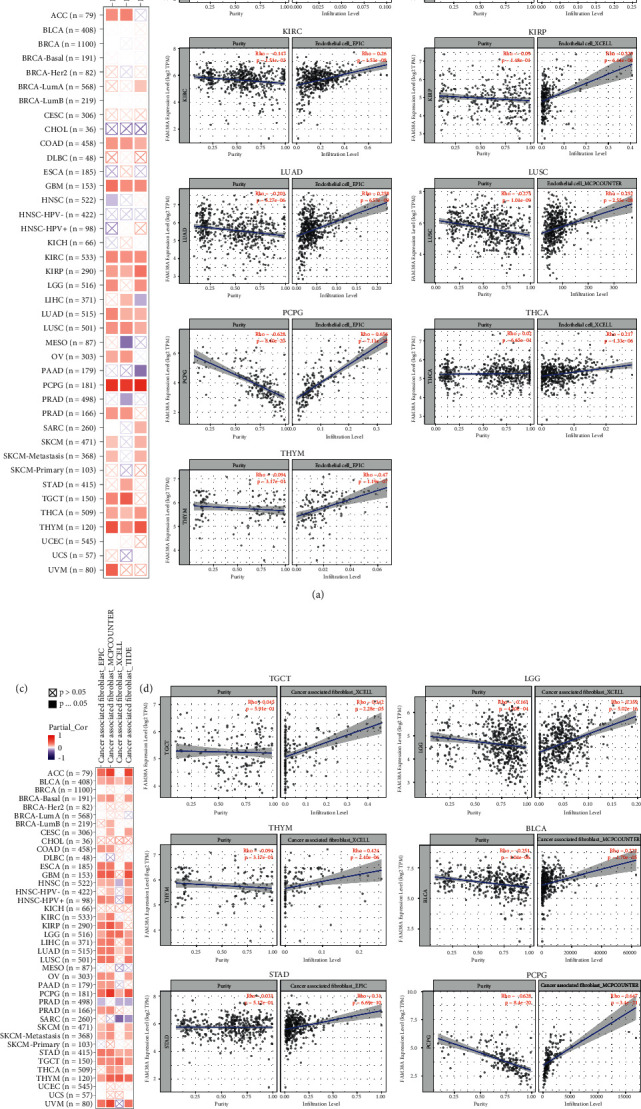
The correlation statistics between PIEZO1 expression and cancer-associated endothelial cells and fibroblasts infiltration. (a-b) The potential associations of the PIEZO1 expression level and infiltration level of cancer association of endothelial cells in various TCGA cancer types assessed by using XCELL, MCP-COUNTER, and EPIC algorithms. Red or blue indicates a positive or negative correlation. (c-d) The potential associations of the PIEZO1 expression level and the level of cancer-association infiltration of fibroblasts in various TCGA cancer types analyzed by using XCELL, MCP-COUNTER, and EPIC algorithms.

Immune infiltration, a major component in the tumor microenvironment, has a crucial effect on the development and prognosis of tumor [24]. The correlation between PIEZO1 expression level and infiltration of immune cells in various tumors was investigated. The data were analyzed through the TCGA database with the XCELL, MCP-COUNTER, EPIC and TIDE algorithms. The results revealed that the expression of PIEZO1 is positively correlated with endothelial cells in COAD (*r* = 0.219, *p* = 0.0003), GBM (*r* = 0.338, *p* < 0.0001), KIRC (*r* = 0.26, *p* < 0.0001), KIRP (*r* = 0.329, *p* < 0.0001), LUAD (*r* = 0.258, *p* < 0.0001), LUSC (*r* = 0.252, *p* < 0.0001), PCPG (*r* = 0.656, *p* < 0.0001), THCA (*r* = 0.217, *p* < 0.0001) and THYM (*r* = 0.47, *p* < 0.0001) (Figures 7(a)) and 7(b)). Meanwhile, the level of PIEZO1 significantly positively associated with cancer-associated fibroblasts in TCGA tumors, including testicular germ cell tumors (TGCT) (*r* = 0.342, *p* < 0.0001), LGG (*r* = 0.359, *p* < 0.0001), thymoma (THYM) (*r* = 0.424, *p* < 0.0001), BLCA (*r* = 0.222, *p* < 0.0001), STAD (*r* = 0.31, *p* < 0.0001) and PCPG (*r* = 0.647, *p* < 0.0001) (Figures 7(c) and 7(d)).

### 3.8. PIEZO1-Related Partners Enrichment Analysis

**Figure 8:**
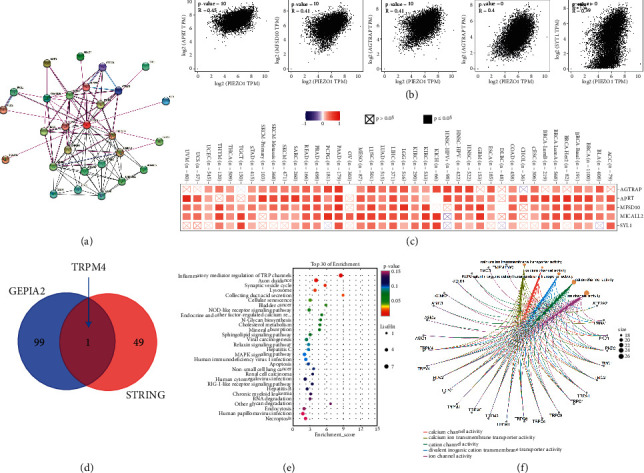
Gene enrichment analysis of PIEZO1. (a) PIEZO1-binding proteins obtained from the STRING tool and displayed through STRING protein network map. (b) By using the GEPIA2 tool, the top 100 PIEZO1-associated genes were obtained. The correlations between PIEZO1 expression and top 5 representative genes (APRT, MFSD10, AGTRAP, MICALL2, and SYTL1) were explored by TCGA and/or GTEx. (c) The expression correlation data between PIEZO1 and APRT, MFSD10, AGTRAP, MICALL2, and SYTL1 in the TCGA tumors shown as heatmap. (d) The intersection of PIEZO1-binding and interacting genes after selection by Venn diagram analysis. (e-f) KEGG and GO enrichment analysis assessed based on PIEZO1-binding and interacted genes.

To reveal the mechanism of the PIEZO1 molecular that links to the oncogenesis of tumors, PIEZO1-binding proteins and pathway enrichment analysis of PIEZO1-correlated genes were performed. 50 PIEZO1-binding proteins by utilizing the STRING tool constructed protein-protein interaction (PPI) networks (Figure 8(a)). Then, we used the GEPIA2 tool to explore the top 100 PIEZO1-associating genes. As assessed in our data, the expression level of PIEZO1 was positively correlated with that of APRT (*r* = 0.45), MFSD10 (*r* = 0.41), AGTRAP (*r* = 0.41), MICALL2 (*r* = 0.4) and SYTL1 (*r* = 0.39) (Figure 8(b)). The heatmap data revealed that the expression level of PIEZO1 was positively correlated with the above five genes in various cancers (Figure 8(c)). Transient receptor potential melastatin 4 (TRPM4), the only one common member in the intersection analysis of the PIEZO1-binding and correlated genes, was showed by the mean of Venn diagram (Figure 8(d)). Then, KEGG and GO enrichment analyses were performed. The KEGG data suggested that “Inflammatory mediator regulation of TRP channels” seem to be associated with the role of PIEZO1 on tumor oncogenesis (Figure 8(e)). The results further showed that these genes are correlated with some activities, such as calcium ion transmembrane transporter activity calcium channel activity, divalent inorganic cation transmembrane transporter activity, cation channel activity and ion channel activity by analyzing GO enrichment data (Figure 8(f)).

### 3.9. PIEZO1 Deletion Reduced the Cell Proliferative Capability

**Figure 9:**
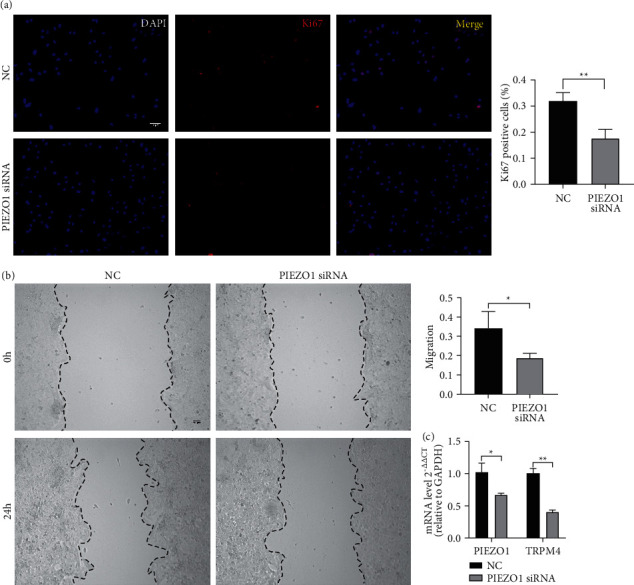
PIEZO1 deletion reduced the cell proliferative capability. (a) PIEZO1 knockdown by PIEZO1 siRNA in CFPAC cell line. Immunofluorescence of ki67 staining was performed, *n* = 3/group. Original magnification x200. Bar = 100 *μ*m. Data are represented as mean ± SD. (b) Scratch assay used to assess migration capability, *n* = 3/group. Original magnification x100. Bar = 100 *μ*m. Data are represented as mean ± SD. (c) The mRNA transcript level of TRPM4 detected by real-time PCR after deletion of PIEZO1 in CFPAC cell line, *n* = 3/group. The bar chart shown represents the mean ± SD. ^*∗*^*P* < 0.05, ^*∗∗*^*p* < 0.01 versus the negative control group by Student's *t*-test.

To further verify the function of PIEZO1, we used PIEZO1 siRNA to knockdown the expression of PIEZO1 in CFPAF cell line. Immunofluorescence staining of ki67 was performed for detecting cell proliferation. The data showed that knockdown of PIEZO1 markedly reduced pancreatic cancer cells proliferation ability (*p*=0.0064) (Figure 9(a)). The cell migration was assessed by scratch assay. The results showed that reduction of PIEZO1 can lower cell migration capacity than their control groups (*p*=0.0420) (Figure 9(b)). In addition, deletion of PIEZO1 can downregulate the expression of TRPM4 (*p*=0.0018) (Figure 9(c)).

## 4. Discussion

PIEZO1 alteration is among the most common genetic alterations in human cancers. Its expression and functions have been studied in different cancer types [25]. It has been considered to have a critical effect on the proliferation, migration and invasion of different tumors [26]. Multiple studies have shown that PIEZO1 may become a reliable diagnostic and prognostic marker in different human cancers. Until now, there are no pancancer studies from the perspective of various cancers. Whether PIEZO1 can affect the pathogenesis of variety types of cancer by some certain molecular pathways remains to be explored. We summarized the features of gene expression, genetic alteration, and protein phosphorylation of PIEZO1 across thirty-three tumors based on the multiple databases, including TCGA, CPTAC, KEGG and GEO.

Our data showed that the expression level of PIEZO1 was significantly increased in BLCA, CHOL, COAD, ESCA, GBM, HNSC, KIRC, LIHC, PAAD, PRAD, READ, STAD, and THCA, but decreased in KICH, KIRP, LUAD, LUSC, PCPG and UCEC compared with adjacent normal tissues. In recent years, studies have confirmed that the expression level of PIEZO1 is significantly increased in gastric cancer [27], esophageal squamous cell carcinoma [28], glioblastoma multiforme [29], colon cancer [25], prostate malignant tumor [9] and bladder carcinoma [30] and decreased in lung cancer. Knockdown of PIEZO1 can inhibit cell migration and tumor growth [10, 31]. This is consistent with our current results. Our immunohistochemistry results revealed that PIEZO1 was downregulated in lung cancer. The different tumors showing different levels of PIEZO1 reflect distinct potential functions and mechanisms.

The data of Kaplan–Meier survival analysis revealed that increasing PIEZO1 expression was correlated with poor OS prognosis in KIRC, LGG, LIHC, LUAD, and LUSC, while in patients with LUAD low PIEZO1 expression was linked with poor OS at the later stage of this disease. However, the high expression level of PIEZO1 correlated with good OS for NSCLC patients, including for patients with LUAD [10]. This is not at par with our current findings. We further discovered that overexpression of PIEZO1 was correlated with worse DFS for patients with ACC, CHOL, GBM, LGG, LIHC and LUSC. Increased expression of PIEZO1 generally predicted poor OS and DFS, which indicated that PIEZO1 is a useful molecular as a prognostic biomarker for patients with tumors.

Cancer is the result of one or more genetic alterations. The way the gene has been altered may determine the prognosis of one cancer [32]. These genetic alterations include gene mutation, structural variants, amplification, deep deletion and multiple alterations. PIEZO1 mostly underwent mutation alteration which is linked to OS, DSS, DFS and PFS in some tumors, such as LIHC, THCA and UCEC. Modification of the PIEZO1 gene could be a promising method to cure cancers. In addition, the PIEZO1 protein phosphorylation level was different between tumor and normal tissues, while different tumors shared diverse phosphorylation sites. These results hint that it is important to clarify the phosphorylation sites of a certain cancer type.

The tumor immune microenvironment (TME) is an integral component of tumor biology [33]. In addition to cancer cells, tumors exhibit another dimension of complexity. They contain a repertoire of recruited, ostensibly normal cells, including smooth muscle cells, endothelial cells, fibroblasts, lymphocytes, macrophages and adipocytes, which significantly contributes to tumor progression [34]. Calcium signaling associated with immune cells plays a key role in regulating cancer development [13–15]. PIEZO1, a mechanosensitive ion channel protein, has identified its effect in the immune system by linking mechanical forces with immune regulation [16–18]. This study evidenced the relationship between the PIEZO1 expression level and the immune infiltration of endothelial cells and cancer-associated fibroblasts in most tumors. The confirmed role of PIEZO1 activity in immune cells raises the possibilities of utilizing PIEZO1 for cancer immunotherapies.

KEGG and GO analyses have revealed the molecular mechanisms of PIEZO1. The results showed that PIEZO1-related genes including APRT, MFSD10, AGTRAP, MICALL2, and SYTL1 were mainly enriched in “Inflammatory mediator regulation of TRP channels.” Our enrichment analyses demonstrated that PIEZO1 may exert its tumorigenic effects by calcium ion transmembrane transporter activity, calcium channel activity, divalent inorganic cation transmembrane transporter activity, cation channel activity, and ion channel activity.

Transient receptor potential melastatin 4 (TRPM4), the only one common member in the intersection analysis of the PIEZO1-binding and correlated genes, was shown by the mean of Venn diagram. TRPM4 is a nonselective cation channel conducting monovalent ions [35]. Intracellular Ca^2+^ directly activates TRPM4 [36]. The protein expression levels of TRPM4 were reported to be increased in various tumors, such as colorectal cancer [37], large B cell lymphoma [38], prostate cancer [39], and so on. The TRPM4 channel also involved in regulating cancer cells to mesenchymal transition, migration and invasion [40]. PIEZO1 enables cells to sense various mechanical forces, which induce the PIEZO1 channel from a closed state turn to an open state. The PIEZO1 channel can allow Ca^2+^ to flow when it is open [41]. However, no study has yet tested the associations between PIEZO1 and TRPM4. Whether the flow of Ca^2+^ caused by PIEZO1 channel affects TRPM4 is unknown. So, we used siRNA to knockdown PIEZO1 and detected the transcriptional level of TRPM4. The result shown that TRPM4 can be downregulated by the deletion of PIEZO1. Of cause, this is only a preliminary exploration. The relationship between the two needs further study.

In conclusion, the statistical correlations of PIEZO1 expression with clinical prognosis, protein phosphorylation and immune cell infiltration across multiple tumors were revealed in our first pancancer analysis in detail, which may promote better understanding of the effect of PIEZO1 in cancerogenesis and provide a novel perspective for cancer immunotherapy.

## Data Availability

The datasets generated and analyzed during the current study are available in the TIMER2 (https://timer.cistrome.org/), GEPIA2 (https://gepia2. cancer-pku.cn/#analysis), UALCAN (https://ualcan.path.uab.edu/analysis-prot.html), ecBioPortal (https://www.cbioportal.org/), and STRING repositories (https://string-db.org/).

## Supplementary Materials

Supplementary Materials The abbreviations and full names of the 33 tumor types were given in Table 1 in supplemental file. In addition, the top 20 related pathways of GSEA were presented in the form of a mountain map (Supplementary Figure S1) for each type of cancer. The results showed that in most tumors, PIEZO1 was associated with the immune responses. These results further illustrated the important role of PIEZO1 in immune regulation.
