# Supplementary material for: A Pancancer Study of PIEZO1 as a Prognosis and Immune Biomarker of Human Tumors
Source: J Oncol. 2022 Jun 14;2022:6725570. doi: 10.1155/2022/6725570 (PMC9213189; doi:10.1155/2022/6725570)
Supplement: Supplementary Materials — The abbreviations and full names of the 33 tumor types were given in Table 1 in supplemental file. In addition, the top 20 related pathways of GSEA were presented in the form of a mountain map (Supplementary Figure S1) for each type of cancer. The results showed that in most tumors, PIEZO1 was associated with the immune responses. These results further illustrated the important role of PIEZO1 in immune regulation. [file 6725570.f1.docx]

Table 1. Abbreviations of pan cancer

| Abbreviations | Full names |
| --- | --- |
| ACC | Adrenocortical carcinoma |
| BLCA | Bladder Urothelial Carcinoma |
| BRCA | Breast invasive carcinoma |
| CESC | Cervical squamous cell carcinoma and endocervical adenocarcinoma |
| CHOL | Cholangiocarcinoma |
| COAD | Colon adenocarcinoma |
| DLBC | Lymphoid Neoplasm Diffuse Large B-cell Lymphoma |
| ESCA | Esophageal carcinoma |
| GBM | Glioblastoma multiforme |
| HNSC | Head and Neck squamous cell carcinoma |
| KICH | Kidney Chromophobe |
| KIRC | Kidney renal clear cell carcinoma |
| KIRP | Kidney renal papillary cell carcinoma |
| LAML | Acute Myeloid Leukemia |
| LGG | Brain Lower Grade Glioma |
| LIHC | Liver hepatocellular carcinoma |
| LUAD | Lung adenocarcinoma |
| LUSC | Lung squamous cell carcinoma |
| MESO | Mesothelioma |
| OV | Ovarian serous cystadenocarcinoma |
| PAAD | Pancreatic adenocarcinoma |
| PCPG | Pheochromocytoma and Paraganglioma |
| PRAD | Prostate adenocarcinoma |
| READ | Rectum adenocarcinoma |
| SARC | Sarcoma |
| SKCM | Skin Cutaneous Melanoma |
| STAD | Stomach adenocarcinoma |
| TGCT | Testicular Germ Cell Tumors |
| THCA | Thyroid carcinoma |
| THYM | Thymoma |
| UCEC | Uterine Corpus Endometrial Carcinoma |
| UCS | Uterine Carcinosarcoma |
| UVM | Uveal Melanoma |

Supplementary Figure S1


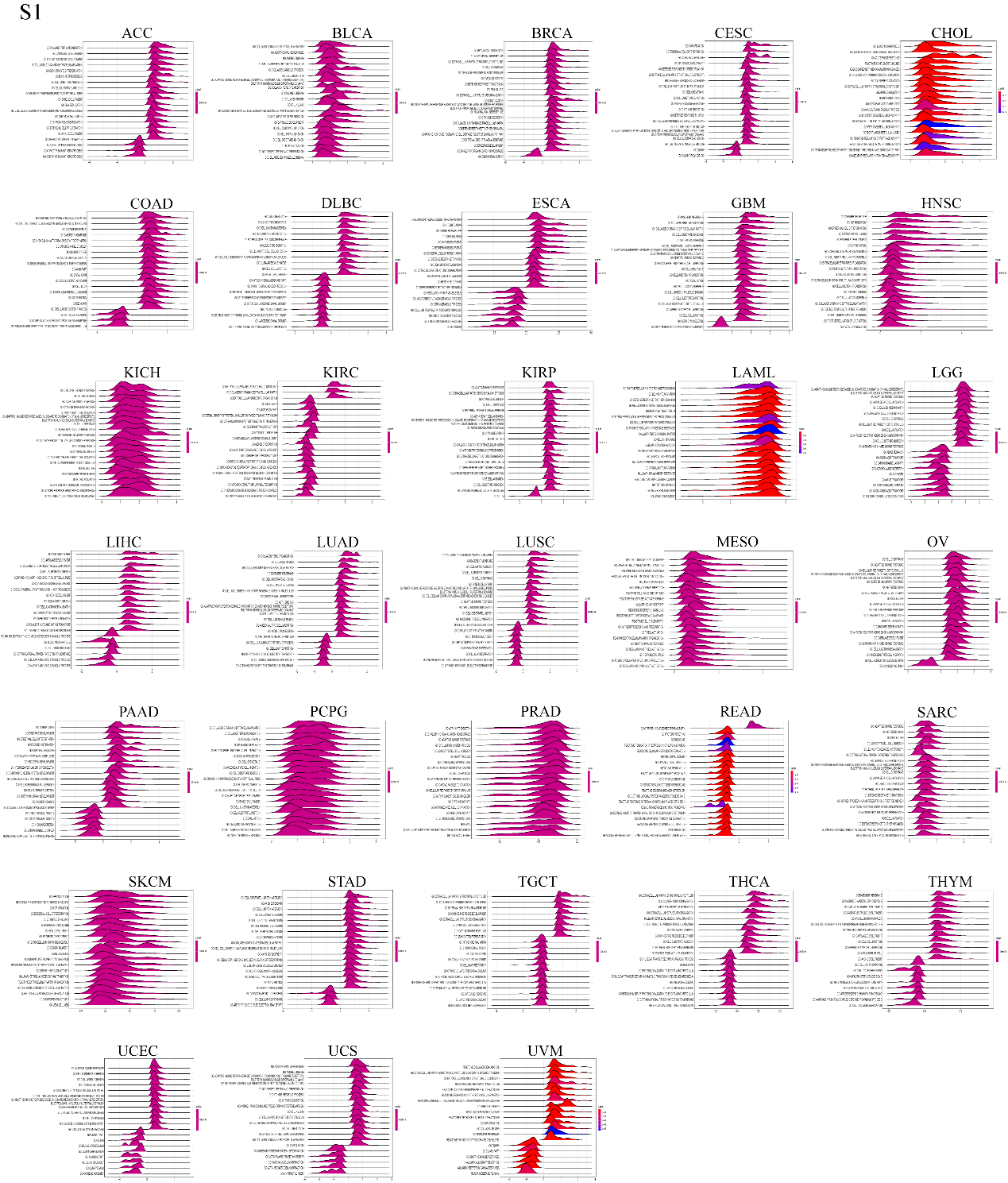


Figure S1. PIEZO1 GSEA results in TCGA pan-cancer. The top 20 related pathways of GSEA are presented in the form of a mountain map.
